# Peripheral blood RNA gene expression in children with pneumococcal meningitis: a prospective case–control study

**DOI:** 10.1136/bmjpo-2017-000092

**Published:** 2017-08-31

**Authors:** Benard W Kulohoma, Fiona Marriage, Olga Vasieva, Limangeni Mankhambo, Kha Nguyen, Malcolm E Molyneux, Elizabeth M Molyneux, Philip J R Day, Enitan D Carrol

**Affiliations:** 1 Centre for Biotechnology and Bioinformatics, University of Nairobi, Nairobi, Kenya; 2 Centre for Integrated Genomic Research, University of Manchester, Manchester, UK; 3 Institute of Integrative Biology, University of Liverpool, Liverpool, UK; 4 Malawi-Liverpool-Wellcome Trust Clinical Research Programme, College of Medicine, Blantyre, Malawi; 5 Institute of Infection and Global Health, University of Liverpool, Liverpool, UK; 6 Department of Paediatrics, University of Malawi, College of Medicine, Blantyre, Malawi

**Keywords:** differential expression, children, invasive pneumococcal disease, host response, pneumococcal meningitis

## Abstract

**Introduction:**

Invasive pneumococcal disease (IPD), caused by *Streptococcus pneumoniae,* is a leading cause of pneumonia, meningitis and septicaemia worldwide, with increased morbidity and mortality in HIV-infected children.

**Objectives:**

We aimed to compare peripheral blood expression profiles between HIV-infected and uninfected children with pneumococcal meningitis and controls, and between survivors and non-survivors, in order to provide insight into the host inflammatory response leading to poorer outcomes.

**Design and setting:**

Prospective case–control observational study in a tertiary hospital in Malawi

**Participants:**

Children aged 2 months to 16 years with pneumococcal meningitis or pneumonia.

**Methods:**

We used the human genome HGU133A Affymetrix array to explore differences in gene expression between cases with pneumococcal meningitis (n=12) and controls, and between HIV-infected and uninfected cases, and validated gene expression profiles for 34 genes using real-time quantitative PCR (RT-qPCR) in an independent set of cases with IPD (n=229) and controls (n=13). Pathway analysis was used to explore genes differentially expressed.

**Results:**

Irrespective of underlying HIV infection, cases showed significant upregulation compared with controls of the following: S100 calcium-binding protein A12 (S100A12); vanin-1 (VNN1); arginase, liver (ARG1); matrix metallopeptidase 9 (MMP9); annexin A3 (ANXA3); interleukin 1 receptor, type II (IL1R2); CD177 molecule (CD177); endocytic adaptor protein (NUMB) and S100 calcium-binding protein A9 (S100A9), cytoskeleton-associated protein 4 (CKAP4); and glycogenin 1 (GYG1). RT-qPCR confirmed differential expression in keeping with microarray results. There was no differential gene expression in HIV-infected compared with HIV-uninfected cases, but there was significant upregulation of folate receptor 3 (FOLR3), S100A12 in survivors compared with non-survivors.

**Conclusion:**

Children with IPD demonstrated increased expression in genes regulating immune activation, oxidative stress, leucocyte adhesion and migration, arginine metabolism, and glucocorticoid receptor signalling.

**What is already known on this topic?**

- Invasive pneumococcal disease (IPD) caused by *Streptococcus pneumoniae* is a leading cause of pneumonia, meningitis and septicaemia worldwide. Globally, IPD is reported to cause about 11% (0.8 million) of all deaths in children less than 5 years of age annually.
- The overall burden of IPD is increased 40-fold in HIV-infected compared with HIV-uninfected children. However, mechanisms involved in host response during IPD are not yet fully understood.

**What this study hopes to add?**

- We demonstrate for the first time differences in transcriptional profiles between HIV-infected and HIV-uninfected children with pneumococcal meningitis (as a homogeneous disease entity of invasive pneumococcal disease and healthy controls).
- We demonstrate increased expression in cases of genes regulating the innate immune response, leucocyte migration, glucose homeostasis and endothelial cell migration.

## Introduction

*Streptococcus pneumoniae* infection is a leading cause of pneumonia, meningitis and septicaemia worldwide, and results in approximately 1 million deaths in children under the age of 5 years annually.^1^ The overall burden of invasive pneumococcal disease (IPD) is increased 40-fold in HIV-infected compared with HIV-uninfected children.^2^ Pneumococcal meningitis is a life-threatening disease with poor prognosis associated with neurologic complications and a high case-fatality ratio in African children, which is further increased by HIV coinfection.^3^

Gene expression profiling during sepsis provides new insights into the host response to invasive bacterial disease. Several sepsis studies have demonstrated upregulation of pathogen recognition receptors and signal transduction pathways, and a downregulation of zinc homeostasis.^4–6^ The intricate host inflammatory response is associated with neuronal and vascular injury, even after cerebrospinal fluid (CSF) sterilisation with antibiotics. Adjunctive new therapies for bacterial meningitis have to date not shown any conclusive benefit, prompting the need for an improved understanding of key mechanisms that might reveal potential new therapeutic targets.^7^ Chronic coinfection may impact gene expression, even among asymptomatic patients. HIV is a major risk factor for IPD, characterised by waning immunity and dysregulated physiology among infected individuals, greatly escalating disease susceptibility and mortality outcomes,^8 9^ thereby influencing the host’s gene expression. We examine differences in gene expression using blood samples from children with pneumococcal meningitis and matched healthy controls, and also compare transcriptional profiles between children with pneumococcal meningitis with and without underlying HIV infection.

## Materials and methods

### Patients and controls

Children (n=377) were recruited as part of a larger study investigating host determinants of susceptibility to IPD conducted at Queen Elizabeth Central Hospital, Blantyre, Malawi, between April 2004 and October 2006.^10^ Ethical approval was granted from the College of Medicine Research Committee, Malawi and the Liverpool School of Tropical Medicine Local Research Ethics Committee. Written informed consent was given prior to recruitment.

We excluded children (n=135) infected with other commonly prevalent microbes (*Salmonella typhimurium*, *Escherichia coli*, *Haemophilus influenzae b*, *Neisseria meningitidis*, *Staphylococcus aureus*, *Streptococcus pyogenes*) identified by positive laboratory culture of blood, CSF or lung aspirate. Cases for the microarray analysis were HIV treatment-naive children with confirmed pneumococcal meningitis defined as abnormal CSF white cell count >10×10^9^/L plus one or more of the following: CSF culture positive for pneumococci, CSF gram stain consistent with pneumococci, CSF positive for pneumococcal polysaccharide antigen or pneumococcal DNA. Cases for the real-time quantitative PCR (RT-qPCR) were children with confirmed IPD, which was defined as follows: pneumococcal pneumonia (n=40) or pneumococcal meningitis (n=189), confirmed by either culture, antigen test or PCR. Controls were healthy afebrile, malaria aparasitaemic children from the same villages as the index cases, and were as closely age matched as possible. Microarray analysis was conducted on 15 samples (12 from cases with pneumococcal meningitis and 3 from controls), and RT-PCR was conducted on 242 samples (229 from cases with IPD and 13 from controls), which included all those used for microarray analysis.

There were no known coinfections apart from HIV, and we did not test for other viruses in the CSF, like cytomegalovirus (CMV) or Epstein Barr Virus (EBV), which have been reported to be associated with increased mortality in Malawian adults with bacterial meningitis.^11 12^

### Samples

Whole blood was drawn at admission from consecutive children with IPD. The methodology for downstream transcriptome analysis from small blood samples has been previously described.^13^

### RNA extraction, quantification and hybridisation

Total RNA was extracted from whole blood using an optimised method for the PAXgene blood RNA kit (Qiagen, West Sussex, UK), as previously described.^13^ The total RNA concentration (ng/µL) and ratios (260/280 and 260/230) were measured using a NanoDrop ND-100 UV-vis spectrophotometer (NanoDrop Technologies, Delaware, USA) and the RNA integrity was assessed using the Agilent 2100 BioAnalyzer (Agilent Technologies, Edinburgh, UK) before and after concentration.

RNA (2 µg) was reverse transcribed into cDNA using the Superscript double-stranded cDNA synthesis kit (Invitrogen, Paisley, UK) according to the manufacturer’s instructions. The double-stranded cDNA was purified using a GeneChip sample clean-up module (Invitrogen). The purified cDNA was then biotin labelled with the ENZO BioArray high-yield RNA transcript labelling kit (Affymetrix, High Wycombe, UK) and cleaned with a cRNA clean-up module (Invitrogen). Aliquots of labelled cRNA (20 µg) were fragmented at 94°C for 35 min and then hybridised to a Human Genome U133A GeneChip array for 16 hours rotating at 60 rpm at 45°C in a GeneChip Hybridization Oven 640 (Affymetrix). Each chip was washed and stained on a GeneChip Fluidics Station 450 (Affymetrix) and scanned using a GeneChip Scanner 3000 (Affymetrix) employing standard recommended protocols (Affymetrix).

### Microarray data analysis

Microarray experiment data were analysed using R and Bioconductor packages.^14^ Briefly, the human genome HGU133A array (Affymetrix) scans output was preprocessed using the affy package.^15^ The limma package was used to evaluate differential expressed genes.^16 17^ Gene annotations were performed using hgu133a.db, KEGG.db database packages.^18–21^ Genes were considered differentially expressed if they had a Benjamini and Hochberg (BH)-adjusted p value <1.5e-3 and >±2-fold change in gene expression. Bonferroni p value adjustments were also performed for comparison. Canonical pathways and functional networks that involve the differently expressed genes play were determined using the Ingenuity Pathway Analysis (IPA) catalogue of well-characterised metabolic and cell signalling cascades. Expression data can be accessed using accession number GSE47172 at the NCBI GEO database.

### Reverse transcription for qPCR

RNA samples were DNAse (Ambion, Warrington, UK) treated to remove any contaminating genomic DNA. RNA (1 µg) was reverse transcribed with SuperScript II RNase H- Reverse Transcriptase and oligo (dT)12–18 (0.5 µg/µL) following the manufacturer’s guidelines. The cDNA was stored at −40°C until required.

### RT-qPCR measurement of target genes

The Human Universal ProbeLibrary (UPL, Roche, Switzerland) employing proprietary locked nucleic acid analogues was used to design qPCR assays to measure expression levels in genes of interest. Using the Roche Online Assay Design Centre, specific primers and an associated probe were selected for the reference and target transcripts. Gene expression was determined using RT-qPCR on a Roche LightCycler 480 (online supplementary table 2).

10.1136/bmjpo-2017-000092.supp1 Supplementary file 1

The following 34 genes were identified from literature and prioritised for RT-qPCR differential expression analysis between cases and controls: ACSL1, ANXA3, ATP, BAG1, BPGM, C3AR1, CA4, CD177, CD55, CD59, CEACAM, CFLAR, FOLR3, GNAI3, GNLY, GYG1, IL1R2, IL1RN, ITGAM, KLRF1, LCK, LCN2, LTF, MAPK14, MMP9, NUMB, OLAH, PSEN1, RETN, S100A12, SAMSN, SERPINA1, SUB1 and VNN1. We used a previously described RT-qPCR data normalisation method.^22^

### Statistical analysis of genes prioritised for RT-qPCR differential expression analysis

First, we derived the relative gene expression in cases compared with controls for the 34 genes under assessment. We used a previously described RT-qPCR data normalisation method.^22^ Briefly, the amounts of target genes expressed in a sample were normalised to the average of the three endogenous controls. This is given by ΔC_q_, where ΔC_q_ is determined by subtracting the average endogenous gene C_q_ value from the average target gene C_q_ value:

C_q_ target gene – C_q_ average endogenous gene = ΔC_q_.

The calculation of relative expression, ΔΔC_q_, involves subtraction of ΔC_q_ value for the controls from the ΔC_q_ value for the cases:

ΔC_q_ target gene_(case)_ – ΔC_q_ target gene_(control)_ = ΔΔC_q_.

2^-ΔΔCq^ is the relative expression of the target gene in cases compared with controls.

Next, we examined statistically significant differences in relative gene expression between cases and controls using the Welch two-sample t-test implemented in the R package. We generate boxplots to visualise the mean and media relative expression in cases and controls separately, and the Welch two-sample t-test p value to show statistically significant differences in relative gene expression between these two groups.

## Results

### Transcriptional profiles among the cases and controls

In the microarray discovery cohort, there were 12 children with pneumococcal meningitis (six male, six female, median age 1.1 years) and 3 controls (two male, one female, median age 7 years). The breakdown was as follows: HIV-infected survivors (n=3), HIV-infected non-survivors (n=3), HIV-uninfected survivors (n=3) and HIV-uninfected non-survivors (n=3) (online supplementary table 1). The RT-PCR validation cohort had a median age of 3.09 years.

10.1136/bmjpo-2017-000092.supp2 Supplementary file 2

**Figure 1:**
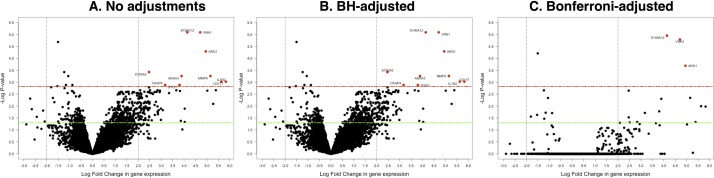
Distribution plot of the differentially expressed genes. The significantly differentially expressed genes are shown in red colour. The significance threshold (p<1.5e-3) is indicated by a dashed red line, and a fold change threshold of more than 2 is shown by the dashed vertical lines. The green line shows p<0.05. (A) Shows results for unadjusted p values, (B) results for BH-adjusted p values and (C) stringent Bonferroni-adjusted p values, which represent an overcorrection. BH, Benjamini and Hochberg.

We examined whether global transcriptional profiles of peripheral blood from children with pneumococcal meningitis (n=12) were distinct from those of healthy controls (n=3) randomly selected from a larger data set by microarray expression profile analysis.^10^ In general, there was a marked distinction in differential expression between cases and controls (online supplementary figure 1). We identified 10 significantly differentially expressed genes (BH-adjusted p value <1.5e-3 and >±2-fold change) (figure 1). We observed significant upregulation of the following: S100 calcium-binding protein A12 (S100A12); vanin-1 (VNN1); arginase, liver (ARG1); matrix metallopeptidase 9 (gelatinase B, 92 kDa gelatinase, 92 kDa type IV collagenase) (MMP9); annexin A3 (ANXA3); interleukin 1 receptor, type II (IL1R2); CD177 molecule (CD177); S100 calcium-binding protein A9 (S100A9), cytoskeleton-associated protein 4 (CKAP4); and glycogenin 1 (GYG1).

10.1136/bmjpo-2017-000092.supp3 Supplementary file 3

### Differential expression, underlying HIV infection and disease outcomes

In the microarray discovery cohort, we did not find any significantly differentially expressed genes for comparisons between HIV-infected cases and HIV-uninfected cases or between survivors and non-survivors (online supplementary figure 2).

10.1136/bmjpo-2017-000092.supp4 Supplementary file 4

### RT-qPCR validation

**Figure 2:**
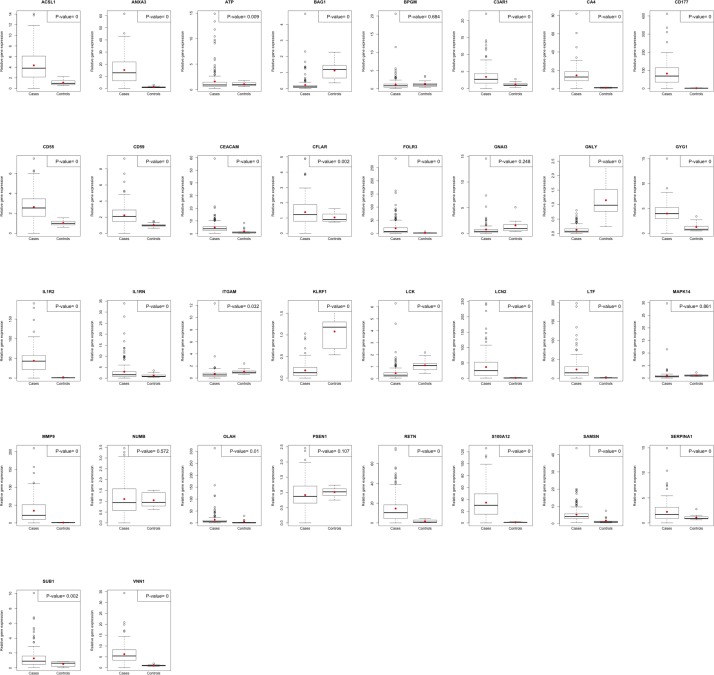
Validation of RNA transcription profile differential expression using real-time quantitative PCR. Relative gene expression in cases compared with controls for 34 genes assessed. The black line shows the boxplot median. The red dot shows the mean, and the Welch two-sample t-test p value is shown on the top right corner.

RT-qPCR validation was performed for a set of 34 prioritised genes selected from literature for cases (n=229) and controls (n=13). The RT-PCR results are in agreement with our microarray analysis findings. All the genes are significantly differentially expressed between the two groups with the exception of guanine nucleotide-binding protein subunit alpha-13 (GNA13), protein numb (NUMB) and presenilin-1 (PSEN1) (figure 2). There was no significantly increased gene expression in HIV-infected compared with HIV-uninfected cases (online supplementary figure 3). Interestingly, there was significant upregulation of folate receptor 3 (FOLR3), NUMB and S100A12 in survivors compared with non-survivors (online supplementary figure 4). There was wide variability in relative gene expression in cases, but not controls.

10.1136/bmjpo-2017-000092.supp5 Supplementary file 5

10.1136/bmjpo-2017-000092.supp6 Supplementary file 6

### Pathway analysis

**Figure 3:**
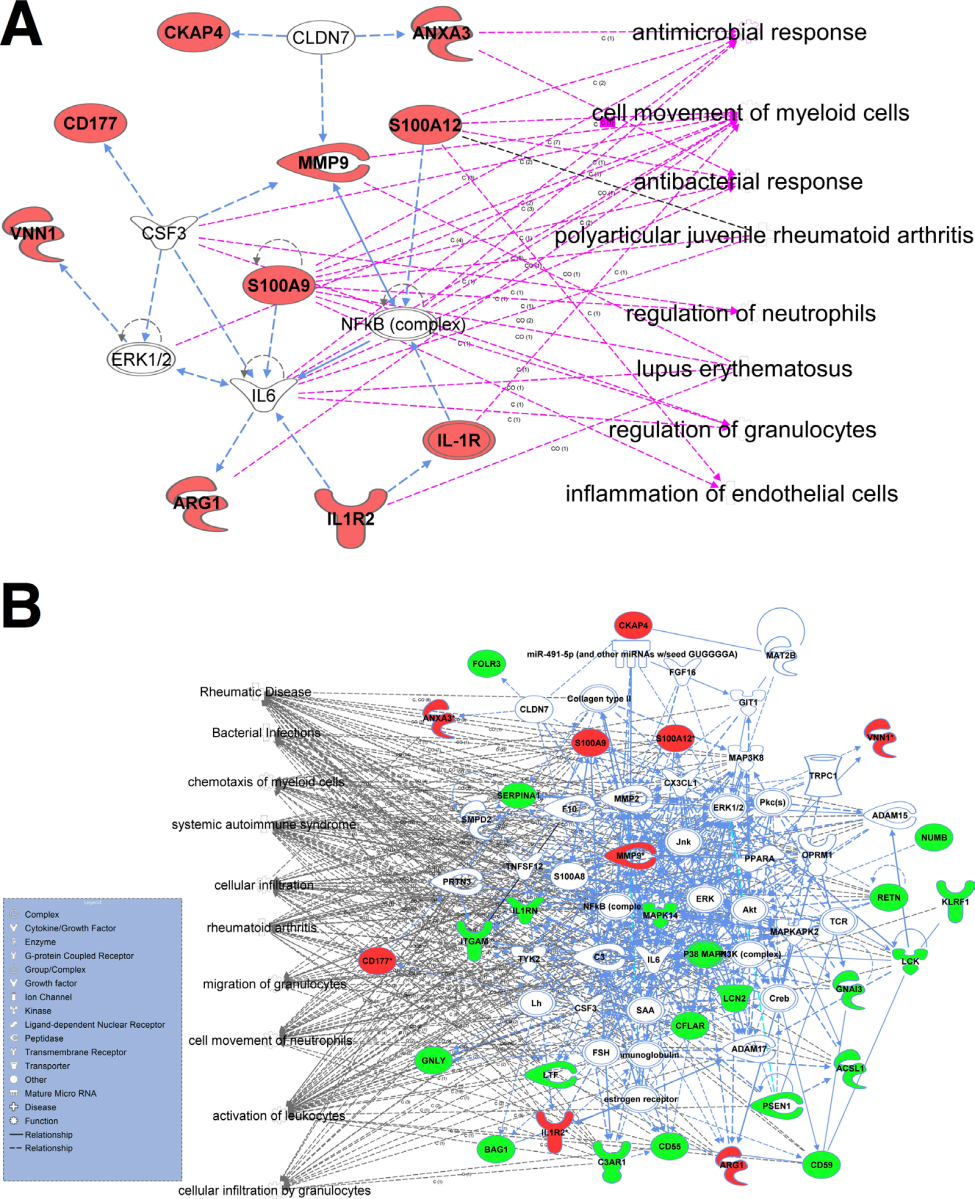
The gene network for significantly differentially expressed genes in the cases. Networks reconstructed to support the most direct connectivity between genes differentially expressed between cases and controls. IPA (Qiagen) software's Core analysis application has been used to perform an automatic graphical reconstruction of the network via utilisation of IPA's Knowledge Base database of protein interactions. The meaning of links and shapes is explained in the inserted legend. Functional connections are presented in blue, information connections to the associated categories in pink and grey. (A) Network reconstructed only for the DE genes identified by microarray analysis. (B) Network reconstructed for the merged data sets of DE genes identified via microarray (red) and PCR analysis (green). White blocks correspond to nodes added by IPA's network editor automatically to ensure network connectivity. DE, differential expression; IPA, Ingenuity Pathway Analysis.

Networks were reconstructed using IPA software for the genes differentially expressed between cases and controls in the microarray experiment, and the combined microarray and RT-qPCR experiments (figure 3A,B). For the genes defined by the microarray experiments, the networks were predominantly related to granulocyte function, and including antimicrobial and endothelial activation responses (figure 3A). The merged set demonstrated wider networks involving immune cell activation as well as leucocyte migration and adhesion (figure 3B). The canonical pathways mapped by genes defined in the microarray experiment demonstrated greatest changes in the arginine and granulocyte pathways. Canonical pathways in the combined microarray and RT-qPCR experiments demonstrated greatest change in leucocytes, and especially neutrophil activation and migration, Notch and glucocorticoid receptor signalling pathways (online supplementary tables 3 and 4).

## Discussion and conclusion

We have shown significant differences in RNA transcriptional profiles in children with pneumococcal meningitis compared with controls. Children with pneumococcal meningitis demonstrated increased expression in genes involved in the inflammatory response, and glucose and L-arginine metabolic pathways. Dysregulation of these pathways can lead to an impaired adaptive host response to pneumococcal infection, thereby contributing to the increased morbidity and mortality. Our findings in the initial cohort of pneumococcal meningitis were validated in the larger cohort of all children with IPD, which includes presentations with pneumonia as well as meningitis. These findings add validity to the initial results from the microarray experiments, and suggest that the host response observed is systemic, and not simply localised to the process of blood–brain barrier disruption per se. We observe wide variability in relative gene expression in cases, which perhaps reflects differences in disease onset and robustness of host response within this group.

Elevation of S100A12, VNN, ARG1, MMP9, ANXA3, IL1R2, CD177, S100A9, CKAP4 and GYG1 in pneumococcal meningitis supports previous findings that have highlighted the important roles of some of these genes during host pathogen response. These genes have important interconnected functions for cellular interactions and response to infection (figure 3A and online supplementary file 7), and play crucial roles during host immune response; cell regulatory processes such as apoptosis and differentiation; and metabolic processes such as amino acid and glucose metabolism.

10.1136/bmjpo-2017-000092.supp7 Supplementary file 7

Vanin-1 (VNN1) protein is expressed by human phagocytes, and involved in leucocyte adhesion and migration.^23^ The VNN1 knockout mice model has provided clear evidence that VNN1 modulates redox and immune pathways.^24^ Exposure of human mononuclear cells to oxidative stress results in upregulation of human VNN1 and downregulation of peroxisome proliferator-activated receptor (PPAR).^25^ IL1R2 and IL1RN were upregulated in cases, which is consistent with our previous report of the IL-1Ra single-nucleotide polymorphism rs4251961 playing a key role in the pathophysiology of IPD and in other human infections.^10^ Recent reports also demonstrate IL1R2 expression upregulation in sepsis, being more pronounced in Gram-negative than Gram-positive infections.^26^

During infection, host production of the cytokine nitric oxide (NO) after non-opsonic phagocytosis exerts microbicidal effects.^27 28^ ARG1 expression is inducible in the lungs of mice in response to pneumococcal infection.^29^ Phagocytosis of pneumococci by macrophages also results in increased production of nitric oxide synthase 2-dependent production of NO and reactive nitrogen species.^30^ Increased plasma arginase activity depletes L-arginine concentrations, the substrate for NO synthesis, leading to vascular dysfunction during severe sepsis and supressed NO-mediated microbicidal effects.^31^ Increased ARG1 activity may also be a bacterial survival strategy to escape the NO-dependent host antimicrobial immune response.^30^

Neutrophil-specific glycoprotein CD177 is expressed on a subset of human neutrophils, and is involved in neutrophil transendothelial migration. A previous microarray study of purified neutrophils from patients with septic shock revealed CD177 mRNA has the highest differential expression between cases and controls.^32^ Consistent with our data, the study also demonstrated increased expression of ARG1, ANXA3, CKAP4, IL1R2, MMP9 and VNN1. ANXA3 promotes endothelial cell junction integrity in animal models, and endothelial cell motility in vitro. ANXA3 is upregulated following neuronal injury, which may explain the finding in pneumococcal meningitis.^33^

S100A12 plays a prominent role in the regulation of proinflammatory processes and immune response by recruiting leucocytes, promoting cytokine and chemokine production, and regulating leucocyte adhesion and migration.^34^ The S100A8/A9 heterodimer is expressed by myeloid cells, especially neutrophils, and has a protective effect in the host response to pneumococcal infection by increasing circulating neutrophils through increased granulocyte colony-stimulating factor production.^35^ It is an antimicrobial peptide, but plays an important role in leucocyte migration.^36^ During infection, S100 proteins stimulate the proinflammatory immune response through interaction with the immunoglobulin family transmembrane pattern recognition receptors: receptor for advanced glycation end-products and Toll-like receptor 4. This leads to nuclear factor kappa B-mediated proinflammatory response with production of proinflammatory cytokines. This inflammatory response in turn leads to increased expression of S100 proteins, and the start of a positive feedback loop. Although as antimicrobial proteins they protect against infection, they can also have a negative detrimental effect on the host by amplifying the destructive proinflammatory responses. The increased expression in survivors may be explained by this positive feedback loop.^37^

Glucocorticoids also play a significant role in immune response regulation by supressing immune and inflammatory responses, and modulating cytokines that promote host immune responses.^38^ We speculate that these responses are important in regulating immune responses to avoid host tissue damage. NUMB negatively regulates Notch, which in turn attenuates proinflammatory cytokines and increases anti-inflammatory cytokines, which may explain the increase in NUMB expression in survivors.^39 40^

Our results are consistent with previous studies on transcription profiling in other bacterial diseases, suggesting that some of the mechanisms are not specific to IPD. Although it could be argued that the lack of age matching in cases versus controls could cause differential expression due to maturation of the immune system in older children, the consistency of our results with other studies makes this unlikely.^41 42^ A limitation of our study is that the microarray analysis was performed using the Affymetrix Human Genome U133A array, and sample number was constrained at the time by cost. The microarray data set was not large enough to allow all possible multifactorial models of comorbidity and disease outcomes to be exhaustively examined, but sought to validate our findings using RT-qPCR in a larger cohort of children. Further evaluation is in a larger cohort using whole genome sequencing would provide more insights on the mechanisms of host response.

In conclusion, this comparative study of gene expression provides mechanistic insight for IPD in children, and demonstrates significant and widespread immune activation, with oxidative stress, recruitment of neutrophils, leucocyte adhesion and migration, activation of antimicrobial peptides and preservation of endothelial cell junction integrity.

10.1136/bmjpo-2017-000092.supp8 Supplementary file 8
