# Supplementary material for: Peripheral blood RNA gene expression in children with pneumococcal meningitis: a prospective case–control study
Source: BMJ Paediatr Open. 2017 Aug 31;1(1):e000092. doi: 10.1136/bmjpo-2017-000092 (PMC5862186; doi:10.1136/bmjpo-2017-000092)
Supplement: Supplementary file 1 [file bmjpo-2017-000092supp001.pdf]

Table S2: Primer sequences and the UPL probe number for each RT qPCR assay

Gene Symbol	Gene	Forward Primer (5'-3')	Reverse Primer (5'-3')	UPL Probe	Accession Number
ACSL1	Acyl-CoA synthetase long-chain family member 1	ccactgtgctgacgttcct	gcactctgtctgtccgtatcc	26	NM_001995
ANXA3	Annexin A3	ttgttaaggaatatcaagcagcat	gccagagagatcacccttca	17	NM_005139
C4A	Complement component 4A (Rodgers blood group)	ggaccctgaagacgaaattg	gaagccctcgttcacctg	75	NM_007293, NM_001252204
C3AR1	complement component 3a receptor 1	aagaaagcaaggcagtccatt	cgtgtgagctcctcactgaa	16	NM_004054
ILR2A	ILR2A interleukin 1 receptor, type II	tacgcaccacagtcaaggaa	aagaaggccagtgaaagtgg	2	NM_004633, NM_173343
MAPK14	MAPK 14 mitogen-activated protein kinase 14	MAPK 14 mitogen-activated protein kinase 14	ccatgagatgggtcaccag	65	NM_139012, NM_139014, NM_001315, NM_139013
MMP9	matrix metallopeptidase 9 (gelatinase B, 92kDa gelatinase, 92kDa type IV collagenase)	gaaccaatctcaccgacagg	gaaccaatctcaccgacagg	6	NM_004994
CD177	CD177 molecule	gcctctccctgatctcctaca	caaggatcctgggtctgc	79	NM_020406
CD55	CD55 molecule, decay accelerating factor for complement (Cromer blood group)	aataatgatgaaggagagtggagtg	tggtgggaccttggaagtta	77	NM_000574
CEACAM1	Carcinoembryonic antigen-related cell adhesion molecule 1 (biliary glycoprotein)	taggcctaagaggctttctcc	tgagggaaagtatgcacagtccgtgtc	80	NM_001712, NM_001184816, NM_001184813, NM_001184815, NM_001205344, NM_001024912
GYG1	Glycogenin 1	ctgaaacagcacaggaccac	atcgccactgtccaagacat	75	NM_004130
FOLR3	Folate receptor 3 (gamma)	cacaaaggctggaattggac	cttgaaggagtggctccaga	19	NM_000804
ITGAM	Integrin, alpha M (complement component 3 receptor 3 subunit)	tctgaagaccattcagaaccag	ggagctgctacttcctgtctg	7	NM_000632
LTF	Lactotransferrin	ctaatctgaaaaagtgctcaacctc	gccatcttcttcggttttacttc	79	NM_002343, NM_001199149
OLAH	Oleoyl-ACP hydrolase	accgagatcgctccactg	ggagaatcaagtgagattttaatagga	19	NM_018324, NM_001039702
RETN	Resistin	tgcaggatgaaagctctctg	catggagcacagggtcttg	45	NM_020415

Gene Symbol	Gene	Forward Primer (5'-3')	Reverse Primer (5'-3')	UPL Probe	Accession Number
S100A12	S100 calcium binding protein A12	tcatatccctggtagccattg	acctactctttgtgggtgtggt	44	NM_005621
SERPINA1	Serpin peptidase inhibitor, clade A (alpha-1 antiproteinase, antitrypsin), member 1	aatggggctgacctctcc	gtcagcacagccttatgcac	82	NM_001002235, NM_001002236, NM_000295
SAMSN1	SAM domain, SH3 domain and nuclear localization signals 1	tgaaaatctgtctgacatggtaca	tagttgggaatgcgtgttca	21	NM_022136, NM_001256370
VNN1	Vanin 1	tcctgaggtgttgctgagtg	agcgtccgtcagttgacac	80	NM_004666
CD59	CD59 molecule, complement regulatory protein	caaggagggtctgtcctgtt	agcactgcaggct[a or g]tgacc	66	NM_000611, NM_203331, NM_203330, NM_203329
CFLAR	CASP8 and FADD-like apoptosis regulator	gcaatccaaaagagtctcaagg	tgagcgccaagctgttcc	7	NM_003879
KLRF1	Killer cell lectin-like receptor subfamily F, member 1	tgatctccttgatcctgttgg	tcttcttgtgccattattcacttt	47	NM_016523
LCN2	Homo sapiens lipocalin 2	tcacctccgtcctgtttagg	aggtaactcgttaatccagggtaa	61	NM_005564
NUMB	Numb homolog (Drosophila)	gaggaagactgatttccccatt	gcacagggagctgatgctc	16	NM_001005745, NM_003744, NM_001005744, NM_001005743
