# Supplementary material for: Peripheral blood RNA gene expression in children with pneumococcal meningitis: a prospective case–control study
Source: BMJ Paediatr Open. 2017 Aug 31;1(1):e000092. doi: 10.1136/bmjpo-2017-000092 (PMC5862186; doi:10.1136/bmjpo-2017-000092)
Supplement: Supplementary file 2 [file bmjpo-2017-000092supp002.pdf]

Table S1: Clinical data on children with pneumococcal meningitis and healthy controls

Sample ID	Age (years)	Sex	Case/control	HIV status	Survival status	Previous antibiotics use	Duration of symptoms (days)
**94**	0.4	F	Case	Positive	Died	No	2
**95**	1.0	F	Case	Positive	Died	Yes	6
**97**	5.7	F	Case	Positive	Died	No	1
**96**	1.2	M	Case	Positive	Alive	No	3
**109**	1.8	F	Case	Positive	Alive	No	2
**123**	12.4	M	Case	Positive	Alive	Yes	3
**82**	13.0	M	Case	Negative	Died	No	7
**115**	0.3	M	Case	Negative	Died	Yes	3
**121**	0.5	F	Case	Negative	Died	No	5
**104**	0.8	M	Case	Negative	Alive	No	3
**105**	0.7	M	Case	Negative	Alive	No	3
**127**	10.6	F	Case	Negative	Alive	Yes	3
**C08**	7.0	M	Control	Negative	Alive	N/A	N/A
**C09**	7.0	M	Control	Negative	Alive	N/A	N/A
**C13**	7.0	F	Control	Negative	Alive	N/A	N/A
