# Supplementary figures and images for: Peripheral blood RNA gene expression in children with pneumococcal meningitis: a prospective case–control study
Source: BMJ Paediatr Open. 2017 Aug 31;1(1):e000092. doi: 10.1136/bmjpo-2017-000092 (PMC5862186; doi:10.1136/bmjpo-2017-000092)

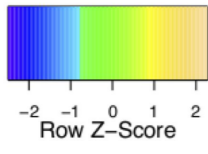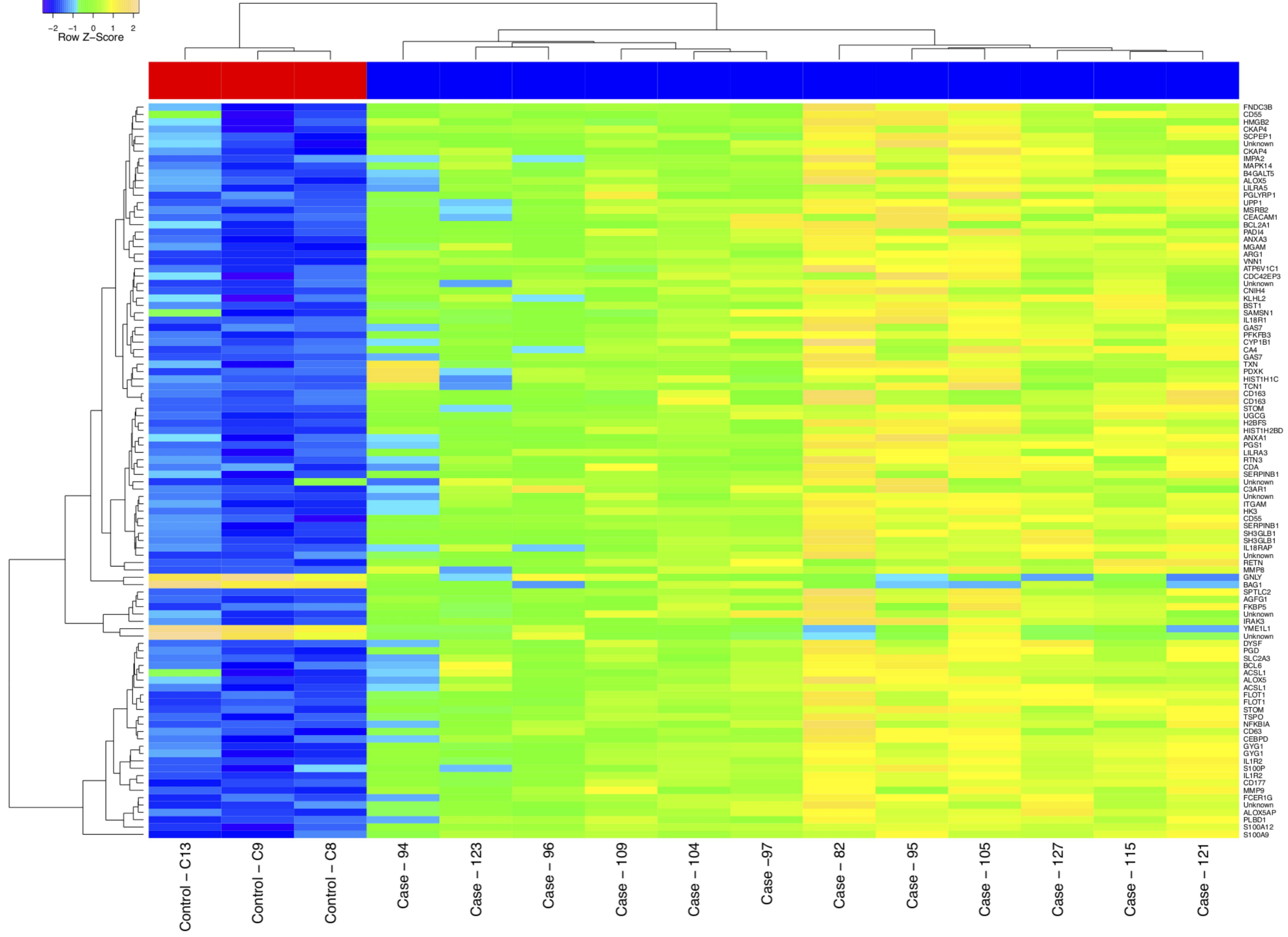

Supplement: Supplementary file 3 [file bmjpo-2017-000092supp003.pdf]

# Survivors vs non-survivors

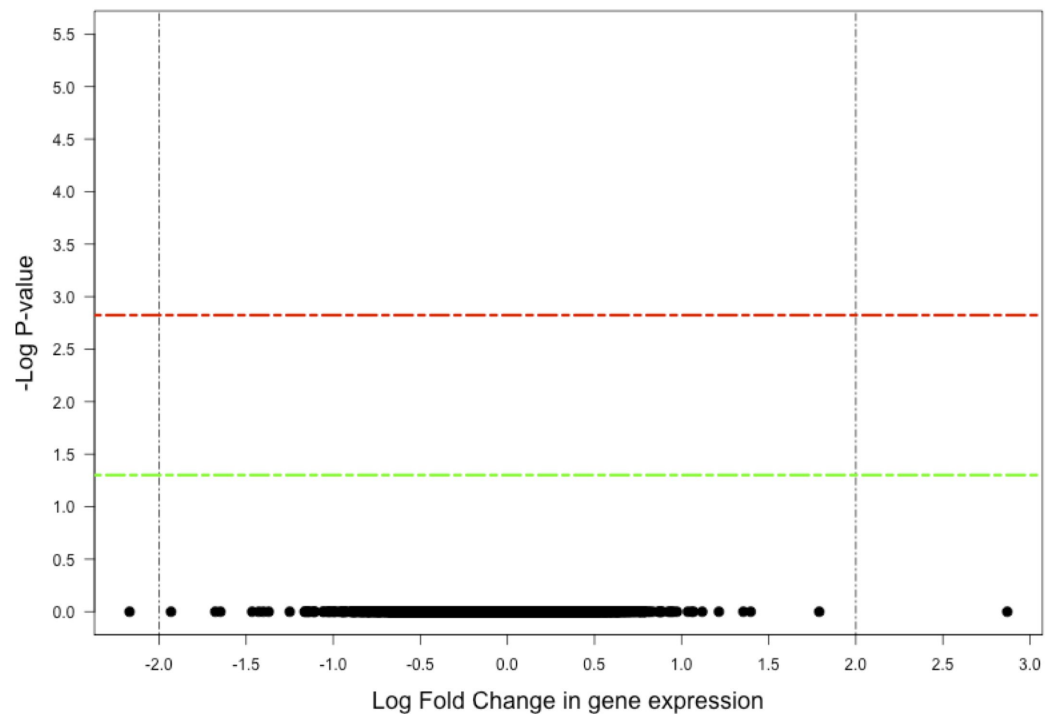

# HIV infected vs HIV un-infected

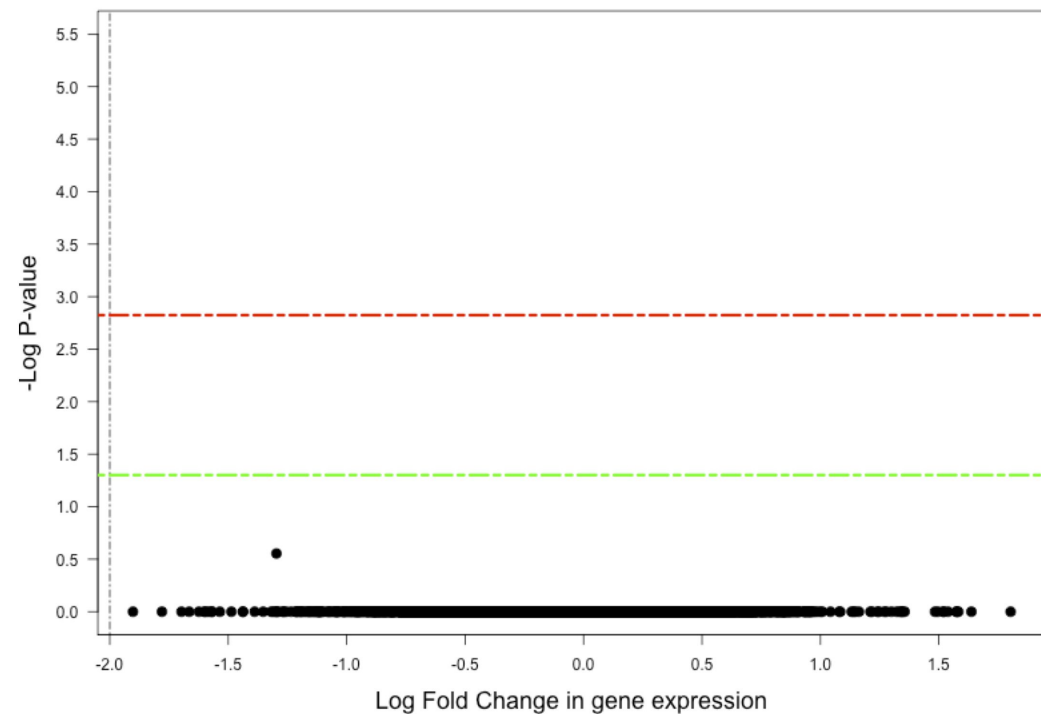

Supplement: Supplementary file 4 [file bmjpo-2017-000092supp004.pdf]

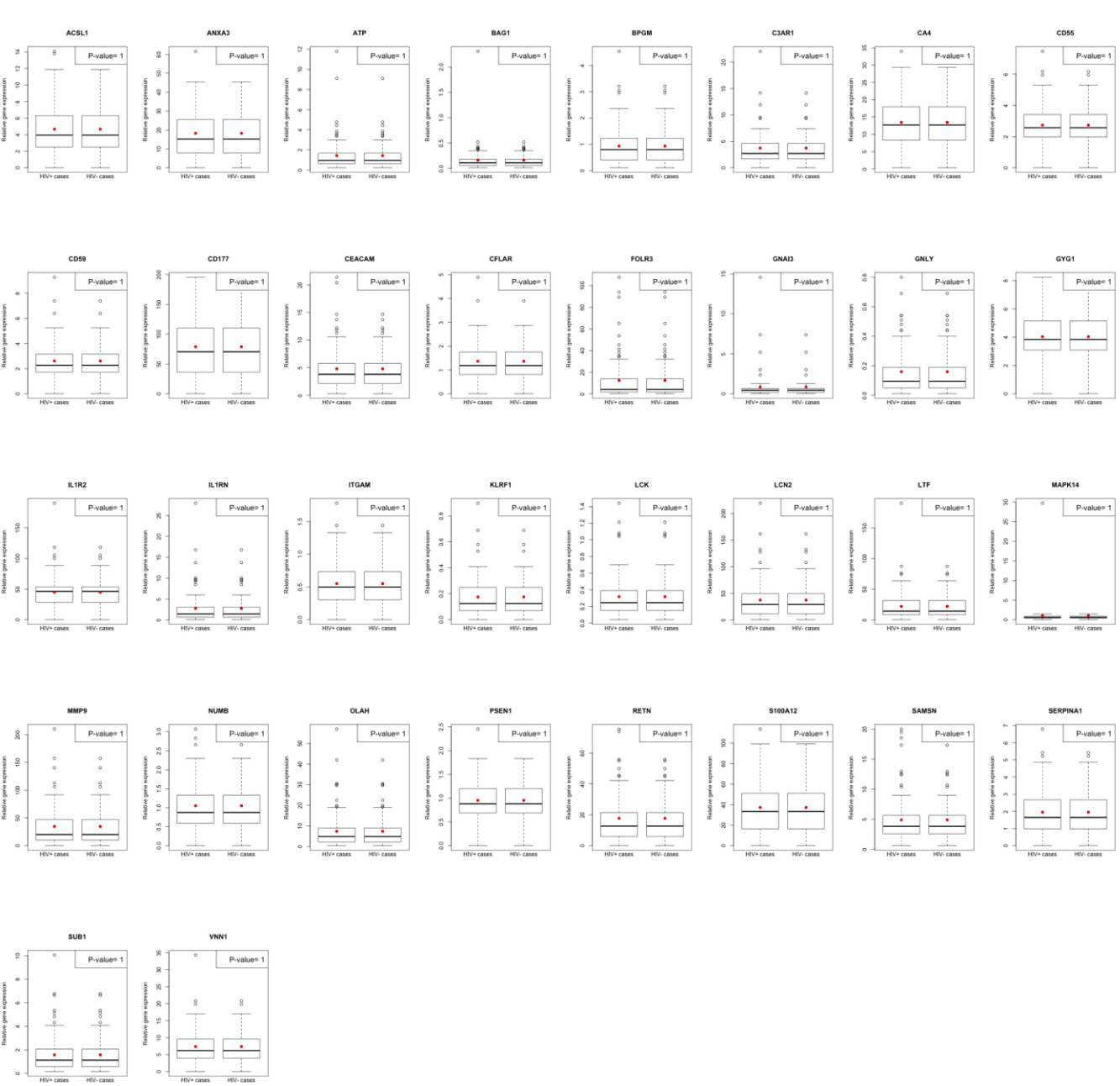

Supplement: Supplementary file 5 [file bmjpo-2017-000092supp005.pdf]

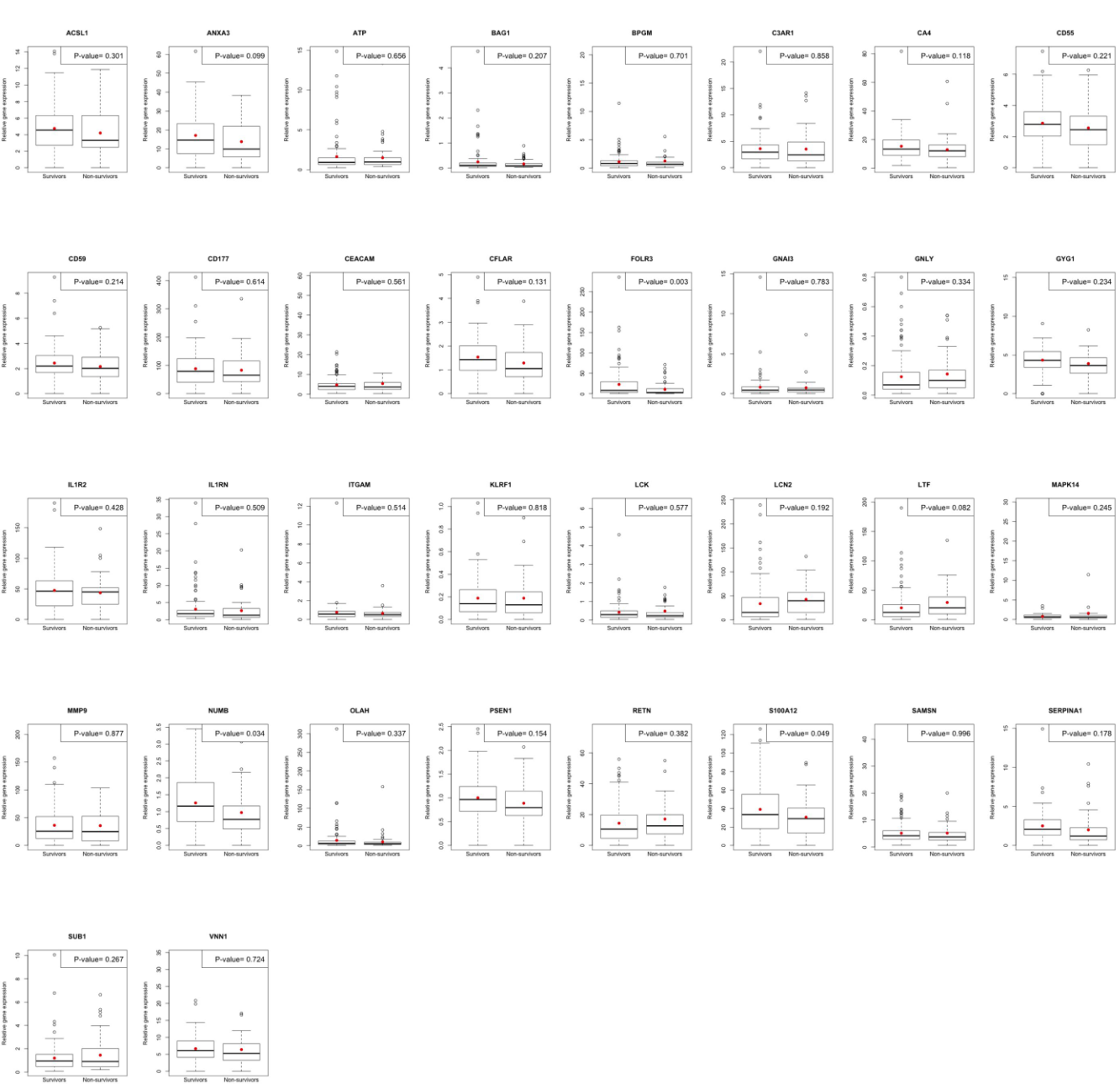

Supplement: Supplementary file 6 [file bmjpo-2017-000092supp006.pdf]
