# Supplementary material for: Peripheral blood RNA gene expression in children with pneumococcal meningitis: a prospective case–control study
Source: BMJ Paediatr Open. 2017 Aug 31;1(1):e000092. doi: 10.1136/bmjpo-2017-000092 (PMC5862186; doi:10.1136/bmjpo-2017-000092)
Supplement: Supplementary file 7 [file bmjpo-2017-000092supp007.pdf]

Supplementary Table 3: Canonical pathways of differentially expressed genes between cases and controls in microarray experiment

Ingenuity Canonical Pathways	-log(p-value)	Ratio	Molecules
LXR/RXR Activation	2.77	0.0165	IL1R2,MMP9
Arginine Degradation I (Arginase Pathway)	2.69	0.25	ARG1
Urea Cycle	2.51	0.167	ARG1
Arginine Degradation VI (Arginase 2 Pathway)	2.51	0.167	ARG1
Glycogen Biosynthesis II (from UDP-D-Glucose)	2.51	0.167	GYG1
Granulocyte Adhesion and Diapedesis	2.51	0.0121	IL1R2,MMP9
Hepatic Fibrosis / Hepatic Stellate Cell Activation	2.43	0.011	IL1R2,MMP9
Airway Pathology in Chronic Obstructive Pulmonary Disease	2.38	0.125	MMP9
Citrulline Biosynthesis	2.38	0.125	ARG1
Role of IL-17A in Psoriasis	2.17	0.0769	S100A9
Superpathway of Citrulline Metabolism	2.14	0.0714	ARG1
Inhibition of Angiogenesis by TSP1	1.79	0.0312	MMP9
Inhibition of Matrix Metalloproteases	1.71	0.0263	MMP9
Neuroprotective Role of THOP1 in Alzheimer's Disease	1.69	0.025	MMP9
IL-10 Signaling	1.46	0.0147	IL1R2
Glioma Invasiveness Signaling	1.45	0.0143	MMP9
Bladder Cancer Signaling	1.36	0.0116	MMP9
PPAR Signaling	1.34	0.0111	IL1R2
HIF1α Signaling	1.25	0.00885	MMP9
p38 MAPK Signaling	1.23	0.00855	IL1R2
Pancreatic Adenocarcinoma Signaling	1.23	0.00847	MMP9
Atherosclerosis Signaling	1.21	0.00806	MMP9
IL-6 Signaling	1.2	0.00787	IL1R2
Ovarian Cancer Signaling	1.15	0.00699	MMP9
Relaxin Signaling	1.13	0.00671	MMP9
Hepatic Cholestasis	1.11	0.00637	IL1R2
PPARα/RXRα Activation	1.08	0.00602	IL1R2
Agranulocyte Adhesion and Diapedesis	1.06	0.00571	MMP9
NF-κB Signaling	1.06	0.00565	IL1R2
Regulation of the Epithelial-Mesenchymal Transition Pathway	1.03	0.00535	MMP9
ILK Signaling	1.02	0.00521	MMP9
IL-8 Signaling	1.01	0.0051	MMP9
Leukocyte Extravasation Signaling	0.996	0.00488	MMP9
LPS/IL-1 Mediated Inhibition of RXR Function	0.99	0.00481	IL1R2
Role of Osteoblasts, Osteoclasts and Chondrocytes in Rheumatoid Arthritis	0.954	0.00441	IL1R2
Colorectal Cancer Metastasis Signaling	0.928	0.00413	MMP9
Glucocorticoid Receptor Signaling	0.862	0.00352	IL1R2
Role of Macrophages, Fibroblasts and Endothelial Cells in Rheumatoid Arthritis	0.84	0.00333	IL1R2
Axonal Guidance Signaling	0.686	0.00226	MMP9
