# Supplementary material for: Peripheral blood RNA gene expression in children with pneumococcal meningitis: a prospective case–control study
Source: BMJ Paediatr Open. 2017 Aug 31;1(1):e000092. doi: 10.1136/bmjpo-2017-000092 (PMC5862186; doi:10.1136/bmjpo-2017-000092)
Supplement: Supplementary file 8 [file bmjpo-2017-000092supp008.pdf]

Supplementary Table 4: Canonical pathways of differentially expressed genes between cases and controls in combined microarray and RT- qPCR experiments

Ingenuity Canonical Pathways	-log(p-value)	Ratio	Molecules
Complement System	6.35	0.111	CD55,CD59,ITGAM,C3AR1
Granulocyte Adhesion and Diapedesis	5.02	0.0303	IL1R2,GNAI3,ITGAM,IL1RN,MMP9
LXR/RXR Activation	4.24	0.0331	IL1R2,IL1RN,SERPINA1,MMP9
IL-10 Signaling	3.64	0.0441	IL1R2,MAPK14,IL1RN
Leukocyte Extravasation Signaling	3.36	0.0195	GNAI3,MAPK14,ITGAM,MMP9
p38 MAPK Signaling	2.95	0.0256	IL1R2,MAPK14,IL1RN
Atherosclerosis Signaling	2.88	0.0242	IL1RN,SERPINA1,MMP9
IL-6 Signaling	2.85	0.0236	IL1R2,MAPK14,IL1RN
Inhibition of Angiogenesis by TSP1	2.84	0.0625	MAPK14,MMP9
Glucocorticoid Receptor Signaling	2.83	0.0141	IL1R2,MAPK14,BAG1,IL1RN
IL-17A Signaling in Fibroblasts	2.77	0.0571	MAPK14,LCN2
Notch Signaling	2.72	0.0541	NUMB,PSEN1
Acute Phase Response Signaling	2.5	0.0179	MAPK14,IL1RN,SERPINA1
Amyloid Processing	2.46	0.04	MAPK14,PSEN1
Agranulocyte Adhesion and Diapedesis	2.45	0.0171	GNAI3,IL1RN,MMP9
NF-κB Signaling	2.44	0.0169	IL1R2,LCK,IL1RN
Molecular Mechanisms of Cancer	2.42	0.0109	GNAI3,MAPK14,CFLAR,PSEN1
IL-8 Signaling	2.31	0.0153	GNAI3,ITGAM,MMP9
LPS/IL-1 Mediated Inhibition of RXR Function	2.24	0.0144	IL1R2,IL1RN,ACSL1
CCR5 Signaling in Macrophages	2.21	0.0299	GNAI3,MAPK14
Chemokine Signaling	2.2	0.0294	GNAI3,MAPK14
Caveolar-mediated Endocytosis Signaling	2.16	0.0282	CD55,ITGAM
Arginine Degradation I (Arginase Pathway)	2.15	0.25	ARG1
Acetate Conversion to Acetyl-CoA	2.15	0.25	ACSL1
Toll-like Receptor Signaling	2.14	0.0274	MAPK14,IL1RN
Role of Osteoblasts, Osteoclasts and Chondrocytes in Rheumatoid Arthritis	2.14	0.0132	IL1R2,MAPK14,IL1RN
IL-15 Signaling	2.1	0.0263	LCK,MAPK14
Rapoport-Luebering Glycolytic Shunt	2.06	0.2	BPGM
PEDF Signaling	2.02	0.0238	MAPK14,CFLAR
Urea Cycle	1.98	0.167	ARG1
Arginine Degradation VI (Arginase 2 Pathway)	1.98	0.167	ARG1
Glycogen Biosynthesis II (from UDP-D-Glucose)	1.98	0.167	GYG1
PPAR Signaling	1.96	0.0222	IL1R2,IL1RN
IL-1 Signaling	1.95	0.022	GNAI3,MAPK14
Cholecystokinin/Gastrin-mediated Signaling	1.87	0.0198	MAPK14,IL1RN
Airway Pathology in Chronic Obstructive Pulmonary Disease	1.86	0.125	MMP9
Citrulline Biosynthesis	1.86	0.125	ARG1
Corticotropin Releasing Hormone Signaling	1.82	0.0187	GNAI3,MAPK14
Role of Macrophages, Fibroblasts and Endothelial Cells in Rheumatoid Arthritis	1.81	0.01	IL1R2,MAPK14,IL1RN
Paxillin Signaling	1.8	0.0182	MAPK14,ITGAM
HIF1α Signaling	1.78	0.0177	MAPK14,MMP9
Role of Tissue Factor in Cancer	1.73	0.0168	LCK,MAPK14
FXR/RXR Activation	1.69	0.016	IL1RN,SERPINA1
CCR3 Signaling in Eosinophils	1.69	0.0159	GNAI3,MAPK14

GNRH Signaling	1.68	0.0157	GNAI3,MAPK14
Role of IL-17A in Psoriasis	1.65	0.0769	S100A9
Fatty Acid Activation	1.65	0.0769	ACSL1
Superpathway of Citrulline Metabolism	1.61	0.0714	ARG1
IL-12 Signaling and Production in Macrophages	1.58	0.0139	MAPK14,SERPINA1
Parkinson's Signaling	1.56	0.0625	MAPK14
Relaxin Signaling	1.55	0.0134	GNAI3,MMP9
γ-linolenate Biosynthesis II (Animals)	1.53	0.0588	ACSL1
Mitochondrial L-carnitine Shuttle Pathway	1.53	0.0588	ACSL1
Oxidative Ethanol Degradation III	1.53	0.0588	ACSL1
Hepatic Cholestasis	1.51	0.0127	IL1R2,IL1RN
PPARα/RXRα Activation	1.47	0.012	IL1R2,MAPK14
Tec Kinase Signaling	1.46	0.0119	GNAI3,LCK
Ethanol Degradation IV	1.44	0.0476	ACSL1
Differential Regulation of Cytokine Production in Intestinal Epithelial Cells by IL-17	1.4	0.0435	LCN2
Role of NFAT in Regulation of the Immune Response	1.4	0.0111	GNAI3,LCK
Hepatic Fibrosis / Hepatic Stellate Cell Activation	1.4	0.011	IL1R2,MMP9
Dendritic Cell Maturation	1.39	0.011	MAPK14,IL1RN
Endothelin-1 Signaling	1.39	0.0109	GNAI3,MAPK14
IL-22 Signaling	1.38	0.0417	MAPK14
Glycolysis I	1.38	0.0417	BPGM
Regulation of the Epithelial-Mesenchymal Transition Pathway	1.37	0.0107	MMP9,PSEN1
IL-17A Signaling in Gastric Cells	1.37	0.04	MAPK14
Role of JAK family kinases in IL-6-type Cytokine Signaling	1.37	0.04	MAPK14
Gluconeogenesis I	1.37	0.04	BPGM
Role of NFAT in Cardiac Hypertrophy	1.36	0.0106	GNAI3,MAPK14
Production of Nitric Oxide and Reactive Oxygen Species in Macrophages	1.35	0.0104	MAPK14,SERPINA1
Clathrin-mediated Endocytosis Signaling	1.33	0.0102	NUMB,SERPINA1
Thrombin Signaling	1.32	0.01	GNAI3,MAPK14
Fatty Acid β-oxidation I	1.29	0.0333	ACSL1
4-1BB Signaling in T Lymphocytes	1.28	0.0323	MAPK14
G Protein Signaling Mediated by Tubby	1.28	0.0323	LCK
Ethanol Degradation II	1.26	0.0312	ACSL1
Systemic Lupus Erythematosus Signaling	1.26	0.00922	LCK,IL1RN
Coagulation System	1.22	0.0286	SERPINA1
Cardiac Hypertrophy Signaling	1.2	0.00862	GNAI3,MAPK14
April Mediated Signaling	1.19	0.0263	MAPK14
Inhibition of Matrix Metalloproteases	1.19	0.0263	MMP9
Role of PKR in Interferon Induction and Antiviral Response	1.17	0.025	MAPK14
B Cell Activating Factor Signaling	1.17	0.025	MAPK14
Neuroprotective Role of THOP1 in Alzheimer's Disease	1.17	0.025	MMP9
Stearate Biosynthesis I (Animals)	1.17	0.025	ACSL1
Role of Hypercytokinemia/hyperchemokinemia in the Pathogenesis of Influenza	1.16	0.0244	IL1RN
UVC-Induced MAPK Signaling	1.15	0.0238	MAPK14
iNOS Signaling	1.14	0.0233	MAPK14
Primary Immunodeficiency Signaling	1.13	0.0227	LCK
Graft-versus-Host Disease Signaling	1.13	0.0227	IL1RN

Role of Cytokines in Mediating Communication between Immune Cells	1.06	0.0192	IL1RN
Retinoic acid Mediated Apoptosis Signaling	1.01	0.0172	CFLAR
MSP-RON Signaling Pathway	1.01	0.0169	ITGAM
Calcium-induced T Lymphocyte Apoptosis	0.999	0.0167	LCK
IL-2 Signaling	0.973	0.0156	LCK
UVB-Induced MAPK Signaling	0.96	0.0152	MAPK14
Role of IL-17A in Arthritis	0.954	0.0149	MAPK14
EGF Signaling	0.948	0.0147	MAPK14
Melatonin Signaling	0.942	0.0145	GNAI3
Glioma Invasiveness Signaling	0.936	0.0143	MMP9
Role of MAPK Signaling in the Pathogenesis of Influenza	0.936	0.0143	MAPK14
ErbB4 Signaling	0.936	0.0143	PSEN1
GPCR-Mediated Integration of Enteroendocrine Signaling Exemplified by an L Cel	0.93	0.0141	GNAI3
Role of PI3K/AKT Signaling in the Pathogenesis of Influenza	0.919	0.0137	GNAI3
Ephrin B Signaling	0.919	0.0137	GNAI3
STAT3 Pathway	0.919	0.0137	MAPK14
BMP signaling pathway	0.913	0.0135	MAPK14
CD40 Signaling	0.897	0.013	MAPK14
IL-17A Signaling in Airway Cells	0.897	0.013	MAPK14
ATM Signaling	0.887	0.0127	MAPK14
FLT3 Signaling in Hematopoietic Progenitor Cells	0.872	0.0122	MAPK14
Communication between Innate and Adaptive Immune Cells	0.872	0.0122	IL1RN
Altered T Cell and B Cell Signaling in Rheumatoid Arthritis	0.867	0.012	IL1RN
GPCR-Mediated Nutrient Sensing in Enteroendocrine Cells	0.862	0.0119	GNAI3
α-Adrenergic Signaling	0.857	0.0118	GNAI3
IL-17 Signaling	0.857	0.0118	MAPK14
Neuregulin Signaling	0.852	0.0116	PSEN1
LPS-stimulated MAPK Signaling	0.852	0.0116	MAPK14
NF-κB Activation by Viruses	0.852	0.0116	LCK
Bladder Cancer Signaling	0.852	0.0116	MMP9
TGF-β Signaling	0.848	0.0115	MAPK14
G Beta Gamma Signaling	0.843	0.0114	GNAI3
Factors Promoting Cardiogenesis in Vertebrates	0.838	0.0112	MAPK14
FGF Signaling	0.834	0.0111	MAPK14
Death Receptor Signaling	0.83	0.011	CFLAR
Reelin Signaling in Neurons	0.825	0.0109	LCK
ErbB Signaling	0.804	0.0103	MAPK14
CTLA4 Signaling in Cytotoxic T Lymphocytes	0.8	0.0102	LCK
CDK5 Signaling	0.8	0.0102	MAPK14
RANK Signaling in Osteoclasts	0.796	0.0101	MAPK14
Antioxidant Action of Vitamin C	0.796	0.0101	MAPK14
UVA-Induced MAPK Signaling	0.788	0.0099	MAPK14
Virus Entry via Endocytic Pathways	0.784	0.0098	CD55
SAPK/JNK Signaling	0.78	0.00971	LCK
Mouse Embryonic Stem Cell Pluripotency	0.772	0.00952	MAPK14
Type I Diabetes Mellitus Signaling	0.769	0.00943	MAPK14
T Cell Receptor Signaling	0.761	0.00926	LCK

Androgen Signaling	0.754	0.00909	GNAI3
p53 Signaling	0.751	0.00901	MAPK14
Axonal Guidance Signaling	0.741	0.00452	GNAI3,MMP9
iCOS-iCOSL Signaling in T Helper Cells	0.73	0.00855	LCK
Pancreatic Adenocarcinoma Signaling	0.726	0.00847	MMP9
Fc Epsilon RI Signaling	0.723	0.0084	MAPK14
Renin-Angiotensin Signaling	0.723	0.0084	MAPK14
Natural Killer Cell Signaling	0.72	0.00833	LCK
fMLP Signaling in Neutrophils	0.72	0.00833	GNAI3
Gαi Signaling	0.72	0.00833	GNAI3
Sphingosine-1-phosphate Signaling	0.713	0.0082	GNAI3
Type II Diabetes Mellitus Signaling	0.704	0.008	ACSL1
CD28 Signaling in T Helper Cells	0.701	0.00794	LCK
PKCθ Signaling in T Lymphocytes	0.698	0.00787	LCK
Cdc42 Signaling	0.692	0.00775	MAPK14
p70S6K Signaling	0.689	0.00769	GNAI3
Th1 Pathway	0.689	0.00769	PSEN1
Role of Pattern Recognition Receptors in Recognition of Bacteria and Viruses	0.686	0.00763	C3AR1
HMGB1 Signaling	0.686	0.00763	MAPK14
P2Y Purigenic Receptor Signaling Pathway	0.686	0.00763	GNAI3
Synaptic Long Term Depression	0.66	0.00714	GNAI3
Ovarian Cancer Signaling	0.652	0.00699	MMP9
Th2 Pathway	0.644	0.00685	PSEN1
Regulation of eIF4 and p70S6K Signaling	0.624	0.00649	MAPK14
Dopamine-DARPP32 Feedback in cAMP Signaling	0.614	0.00633	GNAI3
CXCR4 Signaling	0.6	0.0061	GNAI3
Mitochondrial Dysfunction	0.598	0.00606	PSEN1
Gap Junction Signaling	0.595	0.00602	GNAI3
Germ Cell-Sertoli Cell Junction Signaling	0.589	0.00592	MAPK14
RhoGDI Signaling	0.582	0.00581	GNAI3
Ephrin Receptor Signaling	0.582	0.00581	GNAI3
Sertoli Cell-Sertoli Cell Junction Signaling	0.58	0.00578	MAPK14
Th1 and Th2 Activation Pathway	0.565	0.00556	PSEN1
CREB Signaling in Neurons	0.561	0.00549	GNAI3
B Cell Receptor Signaling	0.561	0.00549	MAPK14
RAR Activation	0.551	0.00535	MAPK14
AMPK Signaling	0.549	0.00532	MAPK14
NRF2-mediated Oxidative Stress Response	0.545	0.00526	MAPK14
ILK Signaling	0.541	0.00521	MMP9
3-phosphoinositide Biosynthesis	0.539	0.00518	LCK
Breast Cancer Regulation by Stathmin1	0.523	0.00495	GNAI3
Integrin Signaling	0.505	0.00472	ITGAM
cAMP-mediated signaling	0.492	0.00455	GNAI3
Superpathway of Inositol Phosphate Compounds	0.481	0.00441	LCK
Phospholipase C Signaling	0.47	0.00427	LCK
Colorectal Cancer Metastasis Signaling	0.458	0.00413	MMP9
Signaling by Rho Family GTPases	0.453	0.00407	GNAI3

Protein Ubiquitination Pathway	0.441	0.00394	BAG1
Xenobiotic Metabolism Signaling	0.422	0.00372	MAPK14
G-Protein Coupled Receptor Signaling	0.42	0.0037	GNAI3
Protein Kinase A Signaling	0.313	0.00266	GNAI3
